# Factors Influencing Wound Healing in Diabetic Foot Patients

**DOI:** 10.3390/medicina60050723

**Published:** 2024-04-27

**Authors:** Sang Heon Lee, Sung Hwan Kim, Kyung Bum Kim, Ho Sung Kim, Young Koo Lee

**Affiliations:** 1 Department of Orthopaedic Surgery, Soonchunhyang University Hospital Bucheon, 170, Jomaru-ro, Wonmi-gu, Bucheon-si 14584, Gyeonggi-do, Republic of Korea; worldking70@naver.com (S.H.L.); shk9528@naver.com (S.H.K.); nine4141@naver.com (H.S.K.); 2 Department of Orthopaedic Surgery, NEW Korea Hospital, 283, Gimpohangang 3-ro, Gimpo-si 10086, Gyeonggi-do, Republic of Korea; ace7106@gmail.com

**Keywords:** diabetic foot, foot ulcer, tracing nutrients

## Abstract

*Background and objectives*: Diabetic foot stands out as one of the most consequential and devastating complications of diabetes. Many factors, including VIPS (Vascular management, Infection management, Pressure relief, and Source of healing), influence the prognosis and treatment of diabetic foot patients. There are many studies on VIPS, but relatively few studies on “sources of healing”. Nutrients that affect wound healing are known, but objective data in diabetic foot patients are insufficient. We hypothesized that “sources of healing” would have many effects on wound healing. The purpose of this study is to know the affecting factors related to the source of healing for diabetic foot patients. *Materials and Methods*: A retrospective review identified 46 consecutive patients who were admitted for diabetic foot management from July 2019 to April 2021 at our department. Several laboratory tests were performed for influencing factor evaluation. We checked serum levels of total protein, albumin, vitamin B, iron, zinc, magnesium, copper, Hb, HbA1c, HDL cholesterol, and LDL cholesterol. These values of diabetic foot patients were compared with normal values. Patients were divided into two groups based on wound healing rate, age, length of hospital stay, and sex, and the test values between the groups were compared. *Results*: Levels of albumin (37%) and Hb (89%) were low in the diabetic foot patients. As for trace elements, levels of iron (97%) and zinc (95%) were low in the patients, but levels of magnesium and copper were usually normal or high. There were no differences in demographic characteristics based on wound healing rate. However, when compared to normal adult values, diabetic foot patients in our data exhibited significantly lower levels of hemoglobin, total protein, albumin, iron, zinc, copper, and HDL cholesterol. When compared based on age and length of hospital stay, hemoglobin levels were significantly lower in both the older age group and the group with longer hospital stays. *Conclusions*: Serum levels of albumin, Hb, iron, and zinc were very low in most diabetic foot patients. These low values may have a negative relationship with wound healing. Nutrient replacements are necessary for wound healing in diabetic foot patients.

## 1. Introduction

Diabetes mellitus (DM) is a chronic metabolic condition that manifests with numerous complications, particularly if it is inadequately controlled. It ranks as the foremost cause of non-traumatic lower limb amputations [1]. Presently, nearly 500 million individuals are believed to be afflicted with DM, with a projected alarming surge in the years to come [2]. Diabetic foot is one of the most significant and devastating complications of diabetes and is defined as a foot affected by ulceration that is associated with neuropathy and/or peripheral arterial disease of the lower limb in a patient with diabetes [3]. Additionally, it is estimated that between one in every three to one in every five individuals with DM will experience a chronic non-healing wound during their lifetime, such as a diabetic foot ulcer. These ulcers have an alarming recurrence rate, with 40% recurring within one year and 65% recurring within five years. Currently, there are no reliable methods available to predict the occurrence of these ulcers [2,4]. Diabetic foot is a common, serious limb-threatening complication and is considered a major cause of morbidity and hospitalization in patients with diabetes [5]. Approximately 15–25% of patients with DM will develop diabetic foot during their lifetime [6].

The pathology of diabetic foot ulcers is exceptionally complex, primarily due to persistent hyperglycemia and associated diabetic complications. These complications include barrier disruption and infection, heightened oxidative stress, neuropathy, microvascular issues, and suboptimal chronic inflammatory responses. Additionally, psychological factors such as the patient’s mental health, self-esteem, and family cohesion can contribute to the complexity of diabetic foot ulcers [7]. Endothelial dysfunction plays a crucial role in diabetes, being closely associated with nitric oxide (NO) deficiency. This deficiency promotes insulin resistance and cardiovascular disease, triggered by the increased production of reactive oxygen species (ROS) mediated by hyperglycemia [8]. Endothelial dysfunction is marked by a prothrombotic state, leading to platelet aggregation, vasoconstriction, and inflammation [9]. In addition to diabetes, endothelial dysfunction is observed in association with hypertension, hyperlipidemia, chronic venous insufficiency, arteriosclerotic cardiovascular disease, smoking, hyperhomocysteinemia (HHcy), aging, and ischemic heart disease [10]. Multiple factors contribute to this issue, including inadequate angiogenesis, heightened oxidative stress, an exacerbated inflammatory response, peripheral neuropathy, and abnormal cell apoptosis [11]. Consequently, there is presently a deficiency of specific targets for interventions aimed at treating diabetic ulcers.

Diabetic foot complications impose a significant physical, psychological, and financial burden on both patients and the community. Over half of patients with diabetic ulcers will experience infection [12]. Additionally, around 85% of all amputations in diabetic patients are preceded by foot ulceration, which can further deteriorate into foot infection or gangrene [13]. Diabetic foot ulcers tend to be deeper and more frequently infected compared to other foot ulcers. This reflects the severe microcirculation ischemia and susceptibility to opportunistic infections, which is a common experience among diabetic individuals [14,15]. Healing a diabetic foot ulcer presents a challenge exacerbated by the high rate of re-ulceration post-healing. Approximately 40% of patients experience the recurrence of an ulcer within one year after healing, nearly 60% within three years, and 65% within five years [16]. Multifactorial approaches are essential to provide affected patients with the best opportunity to heal foot ulcers. Traditionally, this approach has included local wound care, debridement, offloading, meticulous attention to infection control, and, if necessary, revascularization [17].

The diabetic foot ulcer persists as a challenging, recurrent, and sometimes life-threatening complication affecting the lower extremities of individuals with type 2 diabetes mellitus (T2DM). The annual incidence of diabetic foot ulcers is 6.3% for the global population [16]. The heightened incidence of diabetic foot ulcers in patients with DM arises from the interplay of various pathogenic factors, including neuropathy, abnormal foot biomechanics, peripheral arterial disease, and impaired wound healing [7]. Peripheral sensory neuropathy disrupts normal protective mechanisms, enabling patients to sustain major or repeated minor trauma to the foot, often without awareness of the injury. Abnormal weight bearing during walking, resulting from disordered proprioception, can lead to the formation of callus or ulcerations [1]. Both motor and sensory neuropathy contribute to abnormal foot muscle mechanics and structural alterations in the foot, such as hammer toe, claw toe deformity, prominent metatarsal heads, and Charcot joint formation. Autonomic neuropathy leads to anhidrosis and altered superficial blood flow in the foot, which can cause dryness of the skin and the formation of fissures. Peripheral arterial disease and impaired wound healing hinder the resolution of minor skin breaks, leading to their enlargement and susceptibility to infection. Wound healing in diabetic patients is compromised due to both macro- and microvascular diseases, resulting in tissue hypoxia. Additionally, peripheral neuropathy and abnormal cellular and inflammatory pathways contribute to impaired healing, predisposing foot ulcers to infection [18]. Chronic diabetic foot ulcers can be linked to progressive tissue loss, intricate soft tissue and bone infections, and accelerated cardiovascular disease. Approximately 20% of complex, infected diabetic foot ulcers necessitate some form of lower extremity amputation (LEA) [19]. Therefore, it comes as no surprise that a significant portion of patients require lower limb amputations, significantly impacting their quality of life and necessitating costly treatments. The diabetic foot ulcer market alone is projected to escalate from 7.03 billion USD in 2019 to 11.05 billion USD by 2027. Consequently, it is imperative to develop more effective diagnostic and treatment strategies to address this debilitating disease [4,20]. As a result, our capacity to effectively treat early-stage diabetic foot ulcers (ES-DFU) could significantly impact the quality of life and overall survival of individuals experiencing this complication of DM. Impaired wound healing poses a considerable clinical challenge [21] and stands as the primary cause of disability and mortality in diabetic patients who undergo lower extremity amputation [22]. Despite advancements in treatments such as wound debridement, off-loading, medication, wound dressings, and infection prevention through ulcer cleanliness, the persistence of non-healing diabetic foot ulcers remains a significant clinical issue [23]. Wound healing is a complex, dynamic, and interactive process that engages soluble mediators, blood cells, extracellular matrix, and parenchymal cells. It progresses through three overlapping phases: inflammation, tissue formation, and tissue remodeling [24]. Studies have shown that diabetic wound healing is impeded by several factors, including inadequate neoangiogenesis, increased oxidative stress, imbalanced inflammatory response, peripheral neuropathy, and abnormal apoptosis [25]. Indeed, numerous patient-related factors that can influence wound healing are often overlooked, with nutrition being a significant one. The correlation between nutrition and wound healing has been acknowledged for centuries. It is well established that macronutrient deficiencies, particularly in protein, can impede wound healing. Additionally, micronutrients play a crucial role as essential components of cellular metabolism, equally vital for optimal wound healing. Several vitamins and minerals play pivotal roles in supporting the immune system and facilitating wound healing, with vitamin C, vitamin A, and zinc being particularly noteworthy [26]. Moreover, in vitro studies have demonstrated a beneficial interaction between vitamin D and the healing of diabetic foot ulcers [27,28,29]. Some in vivo studies have reported favorable effects of vitamin D in the presence of foot complications. Tiwari et al. demonstrated that vitamin D exerts a beneficial impact on diabetic foot infection [29]. Razzaghi et al. found that, compared to the placebo, vitamin D supplementation over a 12-week period had a significant impact on wound progression in patients with diabetic foot ulcers [30]. Conversely, Afarideh et al. reported no significant difference in vitamin D levels between patients with diabetic foot disease and diabetic patients without foot complications [17]. Despite their significance, these factors are not routinely measured or monitored in clinical practice.

Patients were assessed for VIPS (Vascular supply adequate to heal; absence of clinical signs of deep Infection; Pressure downloading with orthotics and deep-toed shoes; and Special factors) [31]. There are many studies on VIPS, but relatively few studies on “sources of healing”. In particular, there were not many studies related to “nutrition”, mentioned above in the text. Considering the importance of nutrition, this study studied the correlation between various trace elements and wound healing in patients with diabetic foot ulcers.

## 2. Materials and Methods

From July 2019 to April 2021, 46 patients admitted to the orthopedic surgery department with diabetic foot ulcers were enrolled. We measured the blood levels of total protein, albumin, vitamin B12, iron (Fe), zinc (Zn), magnesium (Mg), copper (Cu), hemoglobin (Hb), hemoglobin A1c (HbA1c), high-density lipoprotein (HDL) cholesterol, and low-density lipoprotein (LDL) cholesterol in patients. HbA1c and Hb were measured by the ion exchange high-performance liquid chromatography (HPLC) method [32]. Total protein, albumin, iron, HDL cholesterol, LDL cholesterol, and Mg were measured by spectrophotometry [33]. Vitamin B12 was measured by immunoassay [34]. Zinc and Cu were measured by inductively coupled plasma mass spectrometry [35].

The wound size was measured manually using a ruler in operation rooms. The wound healing rate was measured using the initial wound size and the change of the wound size after 1 week of treatment. Wound healing rate (%) = [(Initial wound size − wound size after 1 week)/Initial wound size] × 100. The wound healing rate was divided into two groups based on a 20% threshold and the differences were compared into two groups. There was no significant difference in age, American Orthopedic Foot and Ankle Society (AOFAS) score, visual analog scale (VAS) score, or length of stay between the two groups (Table 1). Moreover, patients were divided into two groups based on age (60 years, Table 2), length of hospital stay (2 weeks, Table 3), and sex (Table 4), and the investigated parameters between these two groups were compared.

The mean age at presentation was 60.9 years; 33 patients were male (71.7%) and 13 patients were female (28.3%). There were 17 patients with left foot (37%), 23 patients with right foot (50%), and 6 patients with both feet (13%). The patients’ mean AOFAS score was 73.2 and their VAS score was 3.3. Distribution of the VIP was vascular in 15 (32.6%), infection in 28 (60.8%), and pressure in 3 (6.5%) cases.

In healthy adults, using the same test method, 2.5–97.5% was defined as a normal value in the distribution of values. The blood test results of diabetic foot ulcer patients were compared with normal values. Patients were divided by length of hospital stay, age, and sex, and differences among them were also compared.

## 3. Results

Hb was decreased in 91.3% (42 patients) of patients. Total protein was decreased in 19.5% (9 patients) of patients and albumin was decreased in 36.9% (17 patients) of patients. In the case of vitamin B12, it was in the normal range in 71.7% (33 patients), higher than the normal value in 26% (12 patients), and decreased only in 2.1% (1 patients). Among trace elements, iron was decreased in 97.8% (45 patients) of patients and zinc was decreased in 93.2% (41 patients) of patients. Cu was within the normal range in 70.4% (31 patients) of patients, higher than the normal range in 29.5% (13 patients) of patients, and none of the patients were below the normal range. Mg was in the normal range in 84.4% (38 patients) of patients. In the case of cholesterol, HDL cholesterol was lowered in 45.6% of patients (21 patients) and LDL cholesterol was within the normal range in 93.5% (43 patients) of patients (Table 5).

Hb, total protein, albumin, iron, zinc, copper, and HDL cholesterol were lowered compared to the average values of healthy adults. Mg, vitamin B12, and LDL cholesterol were not significantly different (Table 6).

When hospitalized patients were divided into those under 60 years and over 60 years, Hb was measured significantly lower in those over 60, and the length of stay was longer in those over 60 years (Table 2).

When hospitalized patients were divided into those who were hospitalized for less than 2 weeks and those who were hospitalized for more than 2 weeks, Hb and albumin levels were significantly lower in patients hospitalized for more than 2 weeks (Table 3). When hospitalized patients were compared according to sex, vitamin B12 was higher in women, and there was no difference in other values (Table 4).

## 4. Discussion

The facts learned from this study are: that levels of albumin (37%) and Hb (89%) were low in the patients; as for trace elements, levels of iron (97%) and zinc (95%) were low in the patients, but levels of magnesium and copper were usually normal or high; also, glycemic control, as indicated by the mean HbA1c of 7.90 (7.46, 8.35), was generally poor, which is not unexpected.

While hypoglycemia has been linked to vascular complications in diabetes [38], the majority of the literature [39], including this section, concentrates on the adverse impacts of hyperglycemia in relation to the onset and advancement of diabetic foot ulcers. Hyperglycemia plays a role in the development of atherosclerosis, which in turn impedes the delivery of essential nutrients to wounds, thereby hindering the healing process [40]. Additionally, in individuals with DM, hyperglycemia has been identified as a potential factor leading to dysfunction of endothelial cells, which are essential for the healing of diabetic foot ulcers through pressure-induced vasodilation, a response that typically serves as a protective mechanism for the skin [41,42,43]. In addition to endothelial cells, hyperglycemia disturbs essential mechanisms crucial for re-epithelialization, such as the synthesis of proteins, migration, and proliferation of keratinocytes and fibroblasts [44,45]. Another way in which hyperglycemia hampers wound healing is through the generation of free radicals due to decreased activity of antioxidant enzymes such as glutathione peroxidase and superoxide dismutase [46]. This could partly elucidate why other research has indicated that prolonged uncontrolled hyperglycemia correlates with elevated levels of markers related to the skin aging process, specifically advanced glycation end products (AGEs) and their receptors [41]. Hyperglycemia can also induce the production of reactive oxygen species (ROS) through pathways including polyol, hexosamine, protein kinase C, and advanced glycation end products (AGEs) [47]. While reactive oxygen species are necessary for the initial phases of wound healing [48,49], an imbalance in their production has been demonstrated to be detrimental to the later stages of the healing process. In particular, heightened levels of reactive oxygen species can inflict damage on the blood supply, metabolism, and structure of peripheral nerves. This damage to nerves can result in sensory, motor, and/or autonomic dysfunction, with each impairment independently elevating the risk of developing a diabetic foot ulcer [50]. Collectively, these alterations induced by uncontrolled hyperglycemia render the skin more vulnerable to injury and infection, thereby impairing the process of wound healing.

Anemia has been severely reported as a complication of DM [51]. Anemia is associated with poor wound healing, amputation, and increased mortality [31]. Recent studies have shown that anemia is prevalent among patients with DM, particularly in those with diabetic foot ulcers [5,52]. A meta-analysis revealed that the severity of anemia was positively correlated with the severity of diabetic foot ulcers and could potentially serve as a predictor of amputation and mortality [53]. Retrospective cohort studies, including research on 654 and 353 patients with diabetic foot ulcers, have identified anemia as significantly associated with larger, deeper ulcers, more severe infections, higher risk of amputation, and increased mortality rates [54,55], while observational studies conducted in Nigeria have reported an association between anemia and poor wound healing, amputation, and heightened mortality [1,31]. In this study, hemoglobin (Hb) levels were significantly lower in diabetic foot patients compared to healthy individuals, particularly among those over 60 years of age and those hospitalized for 2 weeks or more. Addressing anemia will be crucial for promoting wound healing in diabetic foot patients. For individuals with renal failure, erythropoietin administration may be beneficial. The most common cause of anemia was IDA (iron deficiency anemia) [52]. Iron replacement and therapeutic strategies are recommended to improve their health and quality of life [56]. In this study, iron levels were significantly lowered in DM foot patients compared to healthy people. Iron supply will be important for the treatment of IDA, the main cause of anemia.

Albumin has the ability to maintain the function of endothelial cells [57]. Albumin also improves microcirculatory blood flow and reduces inflammation and oxidative damage [57]. Hypoalbuminemia is a risk factor for wound healing in diabetic foot ulcers [58]. Recent studies of patients with a diabetic foot ulcer showed that serum albumin levels were significantly lower than that in diabetic patients without a diabetic foot ulcer and that low albumin level was an independent predictor for delayed wound healing [57]. Similarly, in this study, albumin levels were significantly lowered in DM foot patients compared to healthy people. Also, the albumin level was measured to be low in patients who were hospitalized for more than 2 weeks. For wound healing, it is thought that albumin level correction and nutrition supply are important [59].

Magnesium (Mg) plays an important role in carbohydrate metabolism as a cofactor of enzymes needed for the phosphorylation of glucose and is also required for glucose transport [60]. Low intracellular levels negatively affect tyrosine kinase activity, glucose transport in cells, and post-receptor insulin action, which, in turn, accentuates hyperglycemia [60]. Reza Razzaghi et al., in their randomized, double-blind, placebo-controlled trial, observed significant benefits in the reduction of ulcer length, width, and depth with Mg supplements for a period of 12 weeks in diabetic foot ulcer patients [61]. A study by Martha Rodríguez-Morán et al. showed a strong relationship between hypomagnesemia and foot ulcers in subjects with type 2 diabetes [62]. Magnesium, which was thought to affect wound healing in previous studies, did not differ between the DM foot patients and healthy people in this study.

Zinc (Zn) is involved in insulin secretion, transport and receptor sensitivity, protection against free radicals, and as a cofactor for enzymes of wound healing [60]. Zinc is the second most abundant trace element in the human body after iron, and its primary sources include animal products and seafood [63]. It is an essential trace element vital for the function of over 300 enzymes and plays a critical role in cellular processes such as cell division and apoptosis [64]. Zinc plays a crucial role in wound healing as it acts as a cofactor in various transcription factors and enzyme systems, including zinc-dependent matrix metalloproteinases. Matrix metalloproteinases constitute a group of calcium-dependent zinc-containing enzymes responsible for degrading the extracellular matrix [65].

Metalloproteinases and their inhibitors play a crucial role in regulating the degradation and deposition of the extracellular matrix during wound repair [66]. Mansooreh Momen-Heravi et al., in their randomized, double-blinded, placebo-controlled trial, observed significant benefits in the reduction of ulcer length and width with zinc supplements for a period of 12 weeks in diabetic foot ulcer patients [67]. The Charu Yadav et al. study showed a statistically significant decrease in the serum levels of zinc in diabetic foot ulcer cases as compared with subjects without ulcers [60]. As with other previous studies, in this study, zinc levels were significantly lowered in DM foot patients compared to healthy people.

Copper (Cu) is essential for the crosslinking of elastin and collagen and mediates angiogenesis via the induction of pro-angiogenic factors, with innate immunity and protection against free radicals [60]. It plays a crucial role in skin regeneration and the formation of new blood vessels, accelerating the healing process by stimulating the production of vascular endothelial growth factor (VEGF) and angiogenesis via the action of hypoxia-inducible factor 1-alpha (HIF-1α) [68]. Charu Yadav et al. noticed a significant decrease in the serum levels of copper (Cu) in the diabetic foot ulcer group [60]. However, in this study, copper in DM foot patients was significantly higher than in healthy people.

Vitamin B12, also known as cobalamin, is a vitamin essential to the proper functioning and development of the central and peripheral nervous systems, ensuring effective nerve-impulse transmission [69]. The Mohammed Badedi et al. study showed that vitamin B12 deficiency was associated with diabetic foot ulcer development in Saudi patients with T2DM in Jazan, Saudi Arabia [70]. However, in this study, there was no significant difference in vitamin B12 levels in DM foot patients compared with healthy people.

High-density lipoproteins (HDL) are circulating particles composed of phospholipids, cholesterol, and proteins. HDL has attracted lots of attention mainly because of its protective effect against the development of atherosclerosis [71]. Lower levels of HDL cholesterol were associated with increased risk for foot ulceration in patients with diabetes (odds ratio 0.427, 95% confidence interval [CI] 0.228–0.799, *p* = 0.008) [72]. No significant associations were found between diabetic foot and LDL cholesterol [73]. In this study, HDL cholesterol was significantly lowered in DM foot patients compared to healthy people. In the case of LDL cholesterol, there was no difference in DM foot patients compared with healthy people.

This study has several limitations. First of all, this is a study conducted at a single institution, and the number of samples is not large. Additionally, long-term research on the effects of trace elements has not been conducted. Based on the results of this study, it was found that trace elements can affect diabetic foot ulcers, and additional research is needed on this. Research conducted at multiple centers and with a larger number of patients is needed, and it is believed that analysis of more factors in addition to the trace elements used in this study will be helpful in the treatment of diabetic foot ulcers. 

## 5. Conclusions

Compared with healthy foot patients, the levels of albumin, Hb, iron, and zinc were low in diabetic foot ulcer patients in our study. In the prognostic assessment and treatment of diabetic foot ulcers, the VIPS classification, which emphasizes vascular, infection, and pressure, as well as nutrition and trace elements as a source of healing, is crucial. Low levels of these parameters can negatively impact wound healing; thus, correction should be considered in the treatment of diabetic foot ulcers.

## Figures and Tables

**Table 1:** Demographic characteristics of patients.

Variables	Wound Healing Rate under 20% (N = 23)	Wound Healing Rate over 20% (N = 23)	*p*-Value
Mean age	61.35 ± 9.01	60.43 ± 7.63	0.7125
Sex			
Female	6	7	
Male	17	16	
Direction			
Left	7	10	
Right	12	11	
Bilateral	4	2	
Mean AOFAS score	71.09 ± 14.80	72.39 ± 14.4	0.7639
Mean VAS score	3.17 ± 1.15	3.35 ± 1.40	0.6482
Length of stay (days)	14.43 ± 11.74	16.13 ± 12.38	0.6359

**Table 2:** Comparison between patients under 60 and over 60.

	Normal Range [33,34]	High	Normal	Low	No. of Case
HbA1C (%)	4–6	43	3	0	46
Hb (g/dL)	13–17	0	4	42	46
Total protein (g/dL)	6.6–8.3	0	37	9	46
Albumin (g/dL)	3.3–5.2	0	29	17	46
Fe (ug/dL)	50–130	0	1	45	46
Mg (ug/dL)	1.5–2.5	3	38	4	45
Zn (ug/dL)	81–121	1	2	41	44
Cu (ug/dL)	70–155	13	31	0	44
Vitamin B12 (pg/mL)	200–950	12	33	1	46
HDL-cholesterol (mg/dL)	45–75	1	24	21	46
LDL-cholesterol (mg/dL)	0–130	3	43	0	46

**Table 3:** Comparison by length of stay.

	Average of Diabetic Foot Ulcer Patients (95% CI)	Normal Values
HbA1C (%)	7.90 (7.46, 8.35)	5
Hb (g/dL)	10.37 (9.73, 11.01)	15
Total protein (g/dL)	6.97 (6.78, 7.15)	7.45
Albumin (g/dL)	3.38 (3.22, 3.54)	4.25
Fe (ug/dL)	35.57 (30.22, 40.91)	125
Mg (ug/dL)	2.14 (2.04, 2.23)	2.15
Zn (ug/dL)	59.90 (55.35, 64.46)	101
Cu (ug/dL)	138.78 (131.17, 146.38)	112.5
Vitamin B12 (pg/mL)	726.78 (558.97, 894.60)	567
HDL-cholesterol (mg/dL)	35.33 (31.84, 38.82)	47.5
LDL-cholesterol (mg/dL)	68.54 (59.52, 77.57)	65

**Table 4:** Comparison by sex.

	Under 60 Years (N = 21)	Over 60 Years (N = 25)	*p*-Value
HbA1C (%)	8.12 ± 1.54	7.72 ± 1.45	0.3765
Hb (g/dL)	11.16 ± 1.77	9.71 ± 2.28	0.0221
Total protein (g/dL)	7.05 ± 0.55	6.89 ± 0.67	0.3849
Albumin (g/dL)	3.53 ± 0.44	3.25 ± 0.58	0.0764
Fe (ug/dL)	40.05 ± 21.14	31.80 ± 14.21	0.1373
Mg (ug/dL)	2.09 ± 0.38	2.17 ± 0.28	0.4084
Zn (ug/dL)	59.62 ± 13.21	60.15 ± 16.97	0.9087
Cu (ug/dL)	137.51 ± 27.95	139.89 ± 23.32	0.7570
Vitamin B12 (pg/mL)	643.19 ± 573.89	797.00 ± 559.51	0.3637
HDL-cholesterol (mg/dL)	37.29 ± 13.03	33.68 ± 10.54	0.3051
LDL-cholesterol (mg/dL)	68.90 ± 36.29	68.24 ± 25.19	0.9439
Length of stay (days)	11.10 ± 4.62	18.80 ± 14.90	0.0205

**Table 5:** Number of patients with high, normal, and low levels of wound healing factors.

	Normal Range [36,37]	High	Normal	Low	No. of Case
HbA1C (%)	4–6	43	3	0	46
Hb (g/dL)	13–17	0	4	42	46
Total protein (g/dL)	6.6–8.3	0	37	9	46
Albumin (g/dL)	3.3–5.2	0	29	17	46
Fe (μg/dL)	50–130	0	1	45	46
Mg (mg/dL)	1.5–2.5	3	38	4	45
Zn (ug/dL)	81–121	1	2	41	44
Cu (ug/dL)	70–155	13	31	0	44
Vitamin B12 (pg/mL)	200–950	12	33	1	46
HDL cholesterol (mg/dL)	45–75	1	24	21	46
LDL cholesterol (mg/dL)	0–130	3	43	0	46

**Table 6:** Average values of diabetic foot ulcer patients and comparison with normal values.

	Less than 2 Weeks (N = 27)	More than 2 Weeks (N = 19)	*p*-Value
HbA1C (%)	7.99 ± 1.73	7.78 ± 1.09	0.6261
Hb (g/dL)	10.98 ± 2.28	9.51 ± 1.71	0.0215
Total protein (g/dL)	7.10 ± 0.51	6.77 ± 0.70	0.0685
Albumin (g/dL)	3.53 ± 0.51	3.16 ± 0.51	0.0195
Fe (ug/dL)	36.44 ± 18.38	34.32 ± 17.84	0.6974
Mg (ug/dL)	2.17 ± 0.32	2.09 ± 0.33	0.4242
Zn (ug/dL)	57.08 ± 12.38	63.77 ± 17.94	0.1458
Cu (ug/dL)	137.62 ± 28.56	140.36 ± 20.71	0.7248
Vitamin B12 (pg/mL)	648.30 ± 513.52	838.32 ± 628.54	0.2662
HDL-cholesterol (mg/dL)	35.78 ± 8.50	34.68 ± 15.50	0.7598
LDL-cholesterol (mg/dL)	73.63 ± 32.32	61.32 ± 26.59	0.1789
Ages	58.93 ± 7.88	63.68 ± 8.20	0.0536

## Data Availability

The original contributions presented in the study are included in the article, further inquiries can be directed to the corresponding author.
